# Impact of layered non-pharmacological interventions on COVID-19 transmission dynamics in Yucatan, Mexico

**DOI:** 10.1016/j.pmedr.2022.101843

**Published:** 2022-05-24

**Authors:** G. Ayora-Talavera, P. Granja-Perez, M. Sauri-Vivas, C.I. Hernández-Fuentes, I.P. Hennessee, I. López-Martínez, G. Barrera-Badillo, A. Che-Mendoza, P. Manrique-Saide, J.A. Clennon, H. Gómez-Dantés, G. Vazquez-Prokopec

**Affiliations:** a Laboratorio de Virología, Centro de Investigaciones Regionales “Dr. Hideyo Noguchi”, Universidad Autónoma de Yucatán, Mérida, Mexico; b Laboratorio Estatal de Salud Pública, Servicios de Salud de Yucatán, Mérida, Mexico; c Servicios de Salud de Yucatán, Mérida, Mexico; d Department of Environmental Health. Rollins School of Public Health. Emory University. Atlanta, GA, USA; e Instituto de Referencia y Diagnóstico Epidemiológicos (InDRE), Secretaría de Salud, México, DF, Mexico; f Campus de Ciencias Biológicas y Agropecuarias, Universidad Autónoma de Yucatán, Mérida, Mexico; g Department of Environmental Sciences, Emory University, Atlanta, GA, USA; h Center for Health Systems Research National Institute of Public Health, Cuernavaca, Mexico

**Keywords:** Non-Pharmacological Interventions, COVID-19, Yucatan, Mexico

## Abstract

**Background:**

The pandemic propagation of SARS-CoV-2 led to the adoption of a myriad of non-pharmacological interventions (NPIs, e.g., social distancing, mobility restrictions, gathering restrictions) in the Americas. Using national epidemiological data, here we report the impact of the layered adoption of multiple NPIs aimed at curving SARS-CoV-2 transmission in Yucatan State, Mexico.

**Methods:**

Data from suspected and laboratory confirmed COVID-19 cases during 2020 were analyzed by age groups and sex, clinical signs, and symptoms as well as outcome. The impact of NPIs was quantified using time-varying reproduction numbers (*R*_t_) estimated as a time-series and by sectors of the city.

**Findings:**

A total of 69,602 suspected cases were reported, 39.3% were laboratory-confirmed. Men were hospitalized (60.2%), more severely ill (3% vs 1.9%) and more likely to die (62%) than women. Early in the outbreak, all sectors in Merida had $R_{t}$ estimates above unity. Once all NPÍs were in place, $R_{t}$ values were dramatically reduced below one, and in the last interval transmission estimates of $R_{t}$ remained below one in all sectors.

**Interpretation:**

In the absence of a COVID-19 vaccination program, the combination and wide adherence of NPÍs led to a low and stable trend in SARS-CoV-2 transmission that did not overwhelm the health sector. Our study reflects that a controlled and planned ease of restrictions to balance health, social and economic recovery resulted in a single wave of transmission that prolonged at low and stable levels.

**Funding:**

GVP received funding from Emory University via the MP3 Initiative.

Research in context **Evidence before the study**

Mexico was the third worst affected country in America by the COVID-19 pandemic. Limited information is available on the implementation of Non-Pharmacological Interventions (NPIs) in Mexico, and the effect of the pandemic on transmissio dynamics at national or regional level.

**Added value of this study**

The implementation of NPIs has significantly affected the evolution of the pandemic worldwide. However, the socioeconomic context of each affected country was the main driver on duration, timing, periodicity, and adherence to NPIs. Mexico was not the exception, and each of the 32 states responded locally to the pandemic with a mix of heterogeneous NPIs. Yucatán State implemented an array of NPIs, including alcohol sales ban and one of the longest and sustained stay-at-home mandates in Mexico. We analyzed the effect of fourteen local and federal NPIs implemented by the Yucatan government at four different intervals of time during the first year of the COVID-19 pandemic. We show that, in Merida (the largest city in Yucatan), the implementation of layered NPIs contributed to an economic recovery within a single wave of transmission at low and stable levels. The Rt indicator was reduced through long sustained adherence to NPIs which helped decrease human mobility and contact patterns throughout the metropolitan area.

**Implications of all the available evidence**

Understanding the COVID-19 pandemic under the context of country socioeconomic and demographics is crucial to minimize the costs of their interventions.

## 1 Introduction

Since its emergence in Wuhan City (Hubei Province, China) in December 2019, the severe acute respiratory syndrome coronavirus (SARS-CoV-2) responsible of the current coronavirus disease (COVID-19) pandemic (Zhu et al., 2020, World Health Organization, 2019) has disrupted societies, economic development, and global public health infrastructure. Early in the pandemic, countries relied on non-pharmacological interventions (NPIs) such as enhanced hand hygiene, respiratory etiquette, use of masks, social distancing, stay-at-home directives, testing, contact tracing, quarantine of suspected cases as well as isolation of confirmed cases, lockdowns, suspension of non-essential activities and cancellation of public events and mass gatherings, travel restrictions, or school closures, to limit the spread of COVID-19 (Ferguson et al., 2020).

The effect of NPIs during the first wave of the pandemic and before vaccination shows differences between nations, geographic regions, and within countries (Liu et al., 2021, Ge et al., 2022). In highly populated nations such as China, United States, India, Brazil or Mexico, with different geographical characteristics, population densities, health status and economical capacity, the efficacy of such interventions, when properly implemented, was high but transient as countries could not sustain the most strict measures such as travel restrictions, suspension of economic activities, or ability to work from home (Xylogiannopoulos et al., 2021, Jones et al., 2020).

In Mexico, the first case of COVID-19 was detected on February 27th, 2020 (Garcés-Ayala et al., 2020). Over the following 18 months, 3.5 millions confirmed cases and an overall mortality of 9% were reported for the country (Conacyt). Initially, infections occurred associated to travelers returning from countries with high local transmission as Italy, Spain, Germany and USA (Worobey et al., 2020, Fernández-Rojas et al., 2021). In response to SARS-CoV-2 introduction, the Federal Ministry of Health established several NPIs in all the 32 states (Oficial, 2020); which consisted on “non-strict” sanitation measures such as reducing physical contact (including no hand shaking), enhanced personal hygiene rules e.g. frequent hand washing, respiratory hygiene, mask mandates and the medical monitoring of detected cases. Community-wide transmission of SARS-CoV-2 was declared on March 23, 2020. This second phase led to the expansion of the initial NPIs to include the suspension of certain non-essential economic activities, the restriction of massive congregations, and the recommendation of home quarantine to the general population. At subnational level, all Mexican states implemented (at variable timing) these mitigation strategies (Knaul et al., 2021); and compared to other Latin American countries, Mexico and Brazil showed a delayed response after the first confirmed case (Touchton et al., 2021).

A national “Healthy distance” campaign (*Jornada de Sana distancia*) was launched by the Mexican federal government on March 24 to promote a personal distance of 1.5 up to 2.25 mts., in addition to the recommendation of preventive home isolation of contacts, quarantine of suspected cases, and the compulsory closure of schools and prohibition of massive public events. On March 30, 2020, a sanitary emergency was declared, and the public health authorities implemented a national lockdown until May 31, 2020. Widespread epidemic transmission was declared by the National government on April 21st of 2020, when some NPIs were lifted (e.g., lockdowns) or partially implemented (Flores-Arguedas et al., 2021). From June 1st and onward, based on the increasing number of cases and hospital admissions, the Health Ministry set up a national epidemiological color-coded risk level (red, orange, yellow and green associated with maximum, high, medium, and low risk, respectively) (de Salud and de Riesgo, 2021) to monitor the epidemiological risk and determine the pace, types, and magnitude of economic activities that could occur on each State (Oficial, 2020, Oficial, 2020).

The efficacy of NPIs at subnational level has been discussed for Mexico, China, US, United Kingdom, Brazil, or Bangladesh, with heterogeneity in the timing of policy implementation to mitigate the spread of COVID-19 emerging as a common trait of pandemic response (Ferguson et al., 2020, Liu et al., 2021, Knaul et al., 2021, Touchton et al., 2021, Masrur et al., 2020). Nonetheless, México and Brazil, two of the Latin American countries with the highest death toll, showed the least stringent national response, compared to other countries from the region.

The burden of COVID-19 in Mexico displayed differences among the 32 states based on social contexts, economic activities, population dynamics, and the individual strategies adopted by each state to cope with the pandemic (Mariano et al., 2021). For instance, in Yucatan State, the local Government and the Ministry of health (MoH) implemented both federal recommendations but also additional local NPIs to limit the effects of the pandemic in the population (Oficial, 2020). The goal of this approach was to minimize the public health and economic impacts of COVID-19 by ‘bending the curve’ of infection while keeping an active economy. This study analyzes and describes the epidemiology of COVID-19 in Yucatan, and the impact of NPIs on COVID-19 reported cases in the capital city of Merida throughout the first year of the pandemic.

## 2 Methods

### 2.1 Data sources

The study is based on the analysis of the national epidemiological surveillance system database (SINAVE) (Secretaria de Salud) from Mexico’s MoH, which provided the data of suspected and laboratory confirmed COVID-19 cases for the state of Yucatan and the capital city of Merida. Clinical data from suspected and confirmed cases, institutional health insurance, and hospital care along with demographic data were obtained from March 1st until December 31st of 2020.

NPIs implemented: In Mexico, federal measures to institute physical distancing or the so-called “*Safe Distance Campaign*” began on March 23, 2020, more than three weeks after the first recorded case in the country (Knaul et al., 2021). On March 14th, public education authorities announced that activities were suspended beginning on the 20th of the same month. On March 24, the official beginning of phase 2, “community transmission”, was declared at the national level, thus suspending non-essential government activities, and reinforcing confinement measures. During the months of March, April, May and the first weeks of June 2020, the national health authorities did not recommend the use of face masks for the general population, despite the evidence suggesting that their use is effective in mitigating contagion. Table 1 shows the description of NPIs and dates of their implementation from official government resources available at National and local level from the Mexican Ministry of Health (https://www.gob.mx/salud), and the Government of Yucatan (https://www.yucatan.gob.mx, https://www.yucatan.gob.mx/gobierno/diario_oficial.php), respectively. Briefly, the *Safe Distance Campaign* initiated in March 24 to April 21st and was extended until May 30, although it continued as a common practice by the population. The campaign was focused to promote safe physical distance (1.5 up to 2.25 mts.) in close and open areas, the basic personal hygiene rules like respiratory hygiene, frequent hand washing, no hand shaking, preventive home isolation of contacts, quarantine of suspected cases, closing schools and non-essential economic activities, and no massive public events. Suspension of non-essential economic activities was highly enforced by Yucatan authorities until September 17th when economic activities were fully reopened. The epidemiologic traffic light risk used colors based on levels of COVID-19 reporting, with red allowing essential economic activities only (the general population is allowed to have walks close to their home), orange extending essential economic activities to some non-essential ones (businesses and public spaces were allowed to work at 30% occupancy but restaurants and other ‘high risk’ places such as schools and gyms remained closed), yellow included all categories of orange, plus opening of restaurants, gyms and bars at reduced (30%) occupancy. Finally, the green light allowed all activities, including returning to school (Table 1). At any level of the epidemiological traffic light, the basic hygiene rules remained as initially implemented (de Salud and de Riesgo, 2021). The first period of alcohol sales ban started on April (10 to 30) and was extended until June 1st. During the month of June alcohol sales were allowed only on a “home delivery service” and during the next two weeks alcohol sales were permitted in all points of sale. From July15^th^ until August 30 alcohol sales were banned again (Oficial, 2020). Face masks mandates in open and close spaces was compulsory since April 4th (del Estado, 2020).

**Table 1:** The non-pharmacological interventions (NPIs) identified and the timeline when they were implemented in the Mexican state of Yucatan.

**Date (2020)**	**NPI**	**Level**	**Interval of analysis**
March 24-to date	Healthy” distance recommendations	National-Local	1
March 24-to date	Closure of schools	National-Local	1
March 30-May 30	1st General suspension of non-essential activities, including bars, restaurants, malls, cinemas, gyms, museums, churches, hotels	National- Local	1
March 20-October 31	1st Local suspension bars, nightclubs, malls, cinemas, gyms, museums, churches, hotels in Yucatan. Restaurants and cafes can open (with sanitary protocols), ,but restricted until 10 pm	Local	1
April 4th-to date	Compulsory use of facemasks in open and close spaces	Local	1
April 10–May 31	1st Alcohol sales prohibition in all establishments and places. Up to 6 years of imprisonment and up to 200 days of fine, in accordance with the State Penal Code.	Local	1
June 1st – To date	Epidemiological Traffic Light	National-Local	2
June 10–17 to July 14	Alcohol restriction. Sales only at authorized shops or home delivered from 12 to 21 hrs. and from Monday to Thursday. Limit: one box with 24 pieces of beer per customer or nine liters, seven “missiles”, or 1.25 L of wine and spirits.	Local	2
July 3rd	Opening of restaurants and recreational businesses only during weekends		2
July 15 –August 31, extended	2nd Alcohol sale prohibition	Local	3
July 15 -September 17	Restriction of mobility (cars). Allowed only from 500 to 2230 hrs.	Local	3–4
September 1st	Alcohol restriction. Sales to domiciles from Monday to Thursday. Opening of restaurants	Local	4
September 1st – December 31	Re-opening non-essential activities including malls, cinemas, gyms, museums, churches	Local	4
September 17th – date	Restriction of mobility (cars). Allowed only from 5.00 to 2330 hrs.	Local	4

### 2.2 Analysis

Trends in COVID-19 for confirmed cases and deaths were analyzed by age groups and sex, clinical signs, and symptoms as well as outcome (ambulatory, hospitalized or death). The impact of NPIs was analyzed for the capital city of Merida which was the main target of the NPIs. Age-specific adjusted incidence rates were estimated by using Yucatan age incidence rates and adjusted with WHO standard populations.

The time-varying or time dependent effective reproduction number or *Rt* is an index of transmissibility which indicates the average number of new infections caused by an infected case in the naive population over any time interval, t, of the epidemic (Fraser and Galvani, 2007). Since the basic reproduction number (Ro) does not measure the effects of public health interventions, we quantify these effects, for every point in time, using the effective reproduction number R(t), a time-dependent metric that changes dynamically in response to community mitigation strategies and political actions (Linka et al., 2020). We estimated $R_{t}$using a method developed by Cori et al. and implemented in the EpiEstim package of R version 4.0.4 (Cori et al., 2013, Donnat and Holmes, 2020). This method uses a Bayesian framework to estimate $R_{t}$ via incident case data and a pre-specified serial case interval, and has been widely used during the COVID-19 pandemic (O’Driscoll et al., 2021, Gostic et al., 2020). We used daily confirmed COVID-19 cases from Mérida and published serial interval estimates from Nishiura et al. (mean = 4.7, standard deviation = 2.9) (Nishiura et al., 2020. Our calculations of $R_{t}$ were done as a time-series for the whole city and as aggregate estimates by sectors of the city. Merida is divided into five health-administration zones of roughly the same population (Center, North, South, East and West). This zone-level analysis was calculated for different periods, compatible with the initiation of different interventions in the city (Table 1). Interval 1 lasted from March 01 – May 30 representing the beginning of the outbreak with the first city-wide ‘safe distance’ intervention and included the closures of schools, and suspension of non-essential activities, including opening of recreational businesses: bars, restaurants, malls, cinemas, gyms, museums, churches, hotels. Interval 2 was from May 31 – July 14 and included on addition, the first alcohol sales prohibition which ended with alcohol sales restriction and the allowance for restaurants only during weekends. Interval 3 lasted from July 15 to August 30 during the implementation of the second alcohol prohibition intervention and the restriction of mobility of cars, prohibited to circulate between 1030 PM and 500 AM (law-enforcement). Interval 4 was from September 1st to December 31 when all interventions were gradually revoked. Zones where $R_{t}$ was significantly different than 1 (p < 0.05) were mapped with dark borders.

### 2.3 Data statement

Data used in this article was derived from administrative health and social data as a secondary use. The original source data is not owned by the researchers and as such cannot be provided to a public repository.

## 3 Results

### 3.1 Epidemiology of COVID-19 in Yucatan State, 2020

The first report of a laboratory-confirmed case of SARS-CoV-2 in Yucatan occurred on March 12, 2020, identified as an imported case from Spain. Fifteen additional imported cases from travelers returning primarily from Europe and USA were laboratory-confirmed as positive to SARS-CoV-2 in the subsequent weeks. Spain was the main source with seven positive cases (44% of all detected early importations) during 2020.

A total of 69,602 suspected cases were reported during the 2020 epidemic, but only 27,387 (39.3%) were laboratory-confirmed and 368 were epidemiologically linked to a confirmed case (0.5%). Around 78% of all cases were registered by the public health sector through the Respiratory Disease Monitoring Health Units (USMER), and most of them (64%) were credited to the MoH of Yucatan (SSY), 12% to the social security institute (IMSS) and 3% to the state health and social security institute (ISSSTE). Yucatan comprises 106 municipalities and Merida, the capital city, accounted for the highest number of suspected and confirmed cases with 61% and 51% of ambulatory and hospitalized confirmed cases, respectively. The cities of Merida, Valladolid, Tizimin, Kanasin, Ticul, Progreso and Uman concentrated 80% of total cases in the state.

Sequence analysis of viruses circulating during the first months of the pandemic showed the predominance of B lineage, with Pangolin sublineages B.1, B.1.1, B.1.234.

Most of the detected COVID-19 cases were managed as ambulatory (81%) and 19% were hospitalized (5,233 cases). In general, men were hospitalized (60.2%), more severely ill (3% vs 1.9%) and more likely to die (62%) than women. Ambulatory services were concentrated on the 19 to 59 age group (85%), while hospital admissions were distributed among the 19 to 59 age group (41%), 70+ years old (31%) and 60 to 69 years old (26%). Positivity rate in ambulatory cases increased from 24% in <5 years old to 46% in the 70-year-old group, while the positivity rate in hospitalized patients was above 60% in the 19 to 59 years group. The rate of positivity also increased as the epidemic developed, and ambulatory positive samples steadily increased until week 30 when a peak of 60% positivity was reached.

A total of 2,272 deaths due to SARS CoV-2 infection were registered during 2020. The older groups aged 70+ contributed with 44% of all deaths, followed by 19 to 59 years (29%) and 60 to 69 years age groups (27%). The pediatric population (18 yr and under) accounted for <1% of COVID-19 deaths. Men in all age groups, contributed with more deaths (>60%) than women except in the 0 to 5 years old group (45%). Lethality rate in hospitalized cases was slightly higher for males (4.4%) than for females (3.4%).

Clinical signs and symptoms of confirmed cases varied among ambulatory and hospitalized patients but very little between men and women. The ambulatory cases showed a wide variety of general and respiratory symptoms. Hospitalized cases tend to show more severe signs and symptoms like chest pain, polypnea, dyspnea, abdominal pain, vomit, and cyanosis. Comorbidities were higher in hospitalized patients: chronic kidney disease (CKD) was 13 times more frequent, chronic obstructive pulmonary disease (8.5), cardiovascular diseases (6.3), immune deficiencies (5.4), diabetes (4.2), high blood pressure (3.1). Other risk factors like obesity and smoking were similar on ambulatory and hospitalized patients (Table 2).

**Table 2:** Clinical signs and comorbidity of confirmed COVID-19 ambulatory and hospitalized patients by sex.

	Ambulatory			Hospitalized			Deaths			
	Women	Men	Total	Women	Men	Total	Women	Men	Total	H/A Ratio*
	N = 10777	N = 11352	N = 22129	N = 2078	N = 3140	N = 5218	N = 849	N = 1423	N = 2,272	
	%	%	%	%	%	%	%	%	%	
Headache	80∙1	77∙6	78∙8	58∙7	56∙4	57∙4	52∙5	54∙5	53∙8	0∙7
Cough	72∙1	72∙2	72∙2	68∙8	76∙7	73∙4	74∙9	79∙8	78	1
Fever	68∙8	72∙8	70∙8	76∙6	80∙9	79∙1	76∙2	81∙9	79∙7	1∙1
Mialgias	56∙4	57	56∙7	43∙7	44∙9	44∙4	40∙3	42∙3	41∙6	0∙8
Arthralgias	44∙9	44∙7	44∙8	33∙4	34∙9	34∙3	32∙7	34∙1	33∙6	0∙8
General malaise	39	38∙6	38∙8	62∙2	63∙5	63	65∙9	64∙3	64∙9	1∙6
Fast Onset	32∙9	34∙6	33∙8	28∙3	28∙1	28∙2	28∙3	28∙1	28∙2	0∙8
Rinorrea	30∙3	27∙1	28∙6	19∙2	18∙4	18∙7	13∙7	16∙9	15∙7	0∙7
Anosmia	22∙5	19∙9	21∙2	8∙7	9∙2	9	8∙7	8∙7	8∙7	0∙4
Disgusia	20∙4	18∙3	19∙4	8∙6	8∙9	8∙8	9∙5	9	9∙2	0∙5
Diarrea	20∙2	20∙6	20∙4	19∙4	19∙4	19∙4	16∙9	14∙4	15∙3	1∙0
Chest pain	16	14∙2	15∙1	28∙5	31∙5	30∙2	29∙7	29∙6	29∙7	2∙0
Obesity	14∙9	13∙6	14∙3	20∙9	16∙4	18∙3	23∙7	16∙7	19∙3	1∙3
Dyspnea	13∙5	13	13∙2	78∙6	83∙4	81∙4	83∙3	83∙5	83∙4	6∙2
High blood pressure	13∙1	12∙2	12∙6	42∙9	37∙3	39∙6	56∙3	45∙1	49∙3	3∙1
Abdominal pain	8∙0	6∙8	7∙4	15∙4	11∙2	12∙9	11∙8	9∙6	10∙4	1∙7
Diabetes	7∙7	7∙2	7∙4	35∙4	28	31∙1	48∙3	33∙2	38∙9	4∙2
Vomit	4∙6	3∙5	4∙0	10∙1	6∙9	8∙2	6∙8	4∙9	5∙6	2∙0
Asma	4∙5	3∙1	3∙8	4∙9	2∙8	3∙7	6	2∙2	3∙6	1∙0
Conjuntivitis	3∙8	3∙7	3∙8	1∙7	2∙5	2∙2	1∙2	1∙9	1∙6	0∙6
Polypnea	3∙2	3∙2	3∙2	37∙2	38∙0	37∙7	36∙6	39∙1	38∙2	11∙8
Smoking	2∙3	5∙5	3∙9	1∙6	6∙4	4∙3	1∙1	0∙6	5∙1	1∙1
Cardiovascular disease	1	1	1	6∙2	6∙5	6∙4	6∙3	6∙8	6∙1	6∙3
Cianosis	0∙9	0∙9	0∙9	3∙8	4∙5	4∙2	4∙6	5∙2	4∙9	4∙6
Inmune deficiencies	0∙6	0∙4	0∙5	2∙7	2∙3	2∙5	5∙4	2∙8	1∙4	5∙4
COPD	0∙6	0∙4	0∙5	5∙7	3∙7	4∙6	8∙5	6∙8	3∙8	8∙5
Chronic kidney disease	0∙6	0∙5	0∙6	8∙7	6∙9	7∙7	13∙2	11∙8	8	13∙2
HIV-AIDS	0∙2	0∙7	0∙4	0∙7	1∙5	1∙1	2∙6	0∙4	0∙7	2∙6

*Hospitalized / Ambulatory ratio.

### 3.2 COVID-19 transmission dynamics in Merida, 2020

**Fig. 1:**
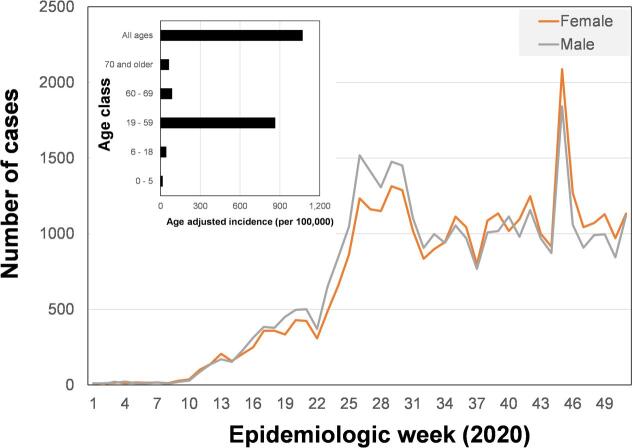
COVID-19 confirmed cases by (A) sex and (B) age specific adjusted incidence rate in Merida, Yucatan, Mexico, during 2020 (expressed as cases/100,000 population).

The epidemic curve of COVID-19 in Merida showed an exponential increase of positive cases in both male and females until week 30, followed by a plateau of around 1,000 cases during 11 consecutive weeks before an abrupt peak recorded in week 45. Despite that, cases fell to previously observed records until the end of the year (Fig. 1A). Age specific adjusted incidence rate of COVID-19 confirmed cases in Merida presented a predominance in the 19 to 39 and 40 to 59 years old age group (Fig. 1B). The trend for men and women in the older age groups (60–69 years and 70+ years old) showed that SARS-CoV-2 was not highly transmitted in this population.

**Fig. 2:**
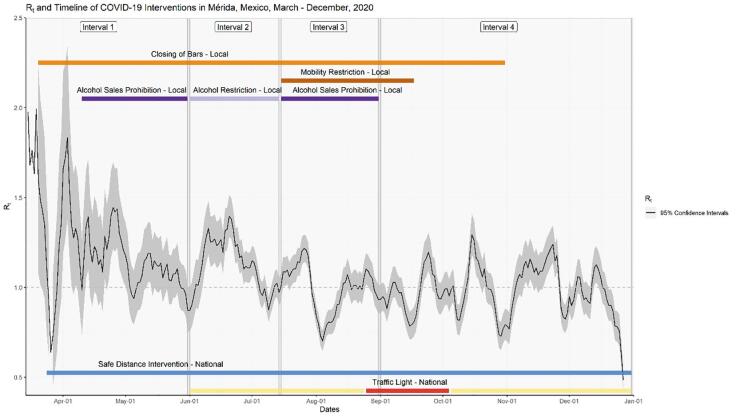
$R_{t}$, Epidemic Curve, and Timeline of COVID-19 Interventions in Mérida, Mexico. March – December 2020.

Fig. 2 shows the daily $R_{t}$ estimates for Merida from the detection of the first case (March 14) until the end of 2020. Early in the introduction of SARS-CoV-2, the estimates of effective transmission were well above one, with 47% of days predicted to have the 95% confidence interval (CI) of $R_{t}$ > 1 during the first interval that only included the general safe distance campaign and suspension of non-essential activities, including opening of recreational businesses. During the second interval, which included the addition of the first alcohol sales prohibition, estimates of $R_{t}$ were slightly reduced, with 43% of days with predicted 95% CI of $R_{t}$ above unity. The third interval, which moved the risk parameters to the ‘orange’ and even to “red” categories with more drastic NPIs between August and September 2020 such as a 2nd alcohol prohibition and reduced mobility, lead to a further reduction in $R_{t}$, which showed evidence of active transmission in 22.7% of the days (Fig. 2). Finally, the fourth interval showed evidence of $R_{t}$ above unity in 25.7% of the days. Overall, the epidemiologic curve and estimates of $R_{t}$ show that Merida did not experience large waves of transmission throughout 2020 (Fig. 2).

**Fig. 3:**
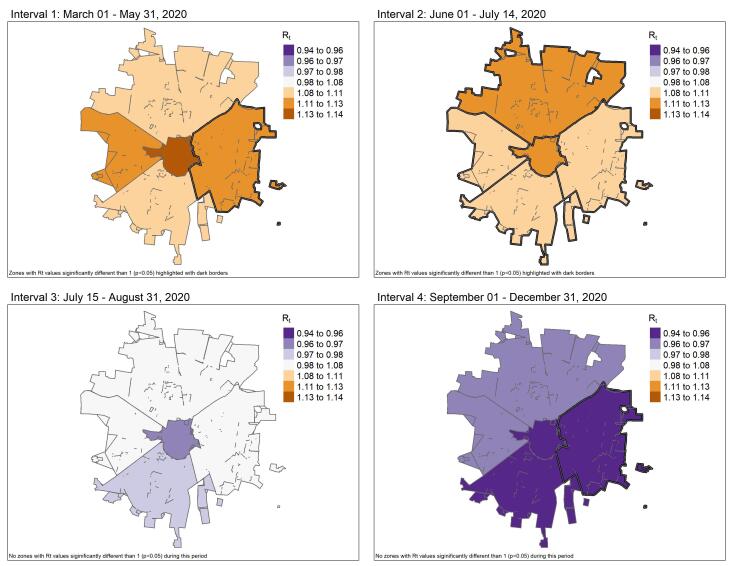
Estimates of $R_{t}$ by Ministry of Health (MOH) sector, split by temporal intervals matching the introduction of different interventions in the city of Mérida, Mexico. March – December 2020.

When $R_{t}$ was calculated by MoH city-sector, a similar trend in reduction of transmission was observed over space–time (Fig. 3). Early in the outbreak (Interval 1), all sectors had $R_{t}$ estimates above unity, with two of them being significantly higher than 1. Such trend increased in interval 2, when four of the five sectors had $R_{t}$ values significantly higher than 1. Once all interventions were in place (interval 3), $R_{t}$ values were dramatically reduced below one, although not significantly different from unity. In the last interval when sustained adherence to NPIs had accumulated, transmission remained low, with estimates of $R_{t}$ being below one in all sectors (Fig. 3).

## 4 Discussion

The current COVID-19 pandemic has impacted every country in the world and Mexico was no exception with 1,851,589 reported cases and 149,169 deaths notified by the national ministry of Health during 2020 and occurring over two ‘waves’ of infection (Conacyt). In Yucatan, we show that the combined adoption of NPIs, and likely increase in human herd immunity, limited SARS-CoV-2 transmission to an initial short-lived wave which never increased above a threshold of 1,000 cases/week in a city of over 1,000,000 inhabitants. The reduction in transmission was associated with a combined implementation of an array of NPIs set in place at different periods. Previous studies (Lai et al., 2020, Li and Zhao, 2020, Tian et al., 2020, Quilty, B.J., Clifford, S., Flasche, S., Eggo, R.M., Group C., 2020, Keeling and Hollingsworth, 2020) have examined the effects of the lockdown, travel restrictions, airport screening, isolation of cases and contact tracing on the containment of the disease, but due to the combination and long-term nature of some NPIs it is important to perform an integrated analysis of its potential impact in the overall epidemic in local settings.

SARS-CoV-2 transmission during 2020 affected all states in Mexico but in different scales in terms of the number of cases, hospitalizations and deaths (Conacyt, Fernández-Rojas et al., 2021, Mariano et al., 2021). Yucatan is among those states where the epidemic did not scale to saturate or surpass the emergency and hospital care facilities. While transmission was identified throughout all the state of Yucatan, Merida the capital city concentrated 61% of ambulatory cases and 51% of hospital admissions. Even though the federal government launched a national campaign to manage the emergence of SARS-CoV-2, Mexican states adopted different strategies according to local contexts and epidemiological realities. NPIs like staying at home, physical distance, no hand shaking, hygienic sneeze, washing hands, and use of face masks were among the most common interventions launched to mitigate transmission and although they were not compulsory, they were adopted by different age groups in different scale and duration.

Yucatan was one of the states with the largest number of regulatory measures in Mexico during 2020 (del Estado, 2020), exceeding national standards in the duration of school closures, and limitation of access to workplaces and public transport, a myriad of public information campaigns, restrictions on internal movements, and international travel but with very similar duration of mask wearing, restrictions on mass gatherings, cancel public events and staying at home measures. To compare performance between states (Knaul et al., 2021), the public policy index is a summary view of state governments’ actions and allows for direct comparisons of how they inform the public, restrict population mobility, maintain public safety, and manage the economic re-opening. Jalisco was the state with the highest average index (52.6), followed by Nuevo Leon (52.2), Nayarit (51.2), Colima (50.0), Sonora (48.7), Yucatan (48.3) and Tamaulipas (47.0). In contrast, the lowest scores in the index during 2020 occurred in Campeche (31.7), Tabasco (38.3), San Luis Potosí (39.0), Quintana Roo (41.0), Guerrero (41.4) and Veracruz (41.4). In the particular case of Yucatan and Merida, -alcohol sales bans or alcohol curfew- was continuous and implemented for the largest period recorded in Mexico (3.5 months split in two periods). This intervention was also imposed in the rest of the country but for much shorter periods than in Merida. Our study could not generate direct evidence of its impact on SARS-CoV-2 transmission (Kianersi et al., 2021), although we speculate that the impact of this ban led to a drastic reduction in social gatherings by the population.

Worldwide, governments deployed a wide range of NPIs and these large-scale interventions were jointly effective at reducing the virus’ effective reproduction number (Soltesz et al., 2020). Across countries, the estimated Rt likely could have been brought below 1 by closing schools and universities, high-risk businesses, and limiting gathering sizes. Business closures and gathering bans were effective at reducing COVID-19 transmission (Brauner et al., 2021) while closing most nonessential face-to-face businesses was not as effective as targeted closures, which only affected businesses with high infection risk, such as bars, restaurants, and nightclubs. Limiting gatherings to 10 people or less was more effective than limits of up to 100 or 1000 people (Liu et al., 2021). Two independent studies concluded that issuing a stay-at home order had a small effect when a country had already closed educational institutions, closed nonessential businesses, and banned gatherings (Brauner et al., 2021, Group C-19 SA). Regardless, COVID-19 has evidenced the need for swift and integrated public health measures to mitigate transmission, and our study provides information that could be used by Yucatan State for developing a coordinated pandemic response plan that can help respond to future pandemics.

The calculation of $R_{t}$ provided additional information about the spatio-temporal pattern of virus transition from an early phase of rapid propagation to a sustained phase of local transmission without any subsequent epidemic waves. This study provides a better understanding of the potential effect of NPIs and evidence for optimizing their use in different settings before natural or vaccine-induced herd immunity are achieved. Estimates of $R_{t}$ by sector in Merida illustrated how transmission dispersion cease to be important by the third interval from July to September, with case counts remaining low in the following months. The combination and wide adherence of case-based and population-based interventions may explain the success of COVID-19 control in Merida throughout 2020 as it has also been demonstrated in other countries (Scarpetta et al., 2021), but it is clear that either category of interventions alone would have been insufficient. As reported in Kermanshah province, Iran (Najafi et al., 2020), the low reproduction number for COVID-19 is an indication of the effectiveness of preventive and intervention programs such as quarantine and isolation. In mainland China the effectiveness of different interventions varied but early detection and isolation of cases prevented more infections than did travel restrictions and contact reductions, and the combination of non-pharmaceutical interventions achieved the strongest and most rapid effect (Lai et al., 2020).

Following one month of social distancing and lockdown, the reproduction number decreased from 2.2 to 1.6 in Hubei Province, China (Li et al., 2020, Zhang et al., 2020); and its decline in most of the United States suggested that social isolation measures may be having a beneficial effect. The premature lifting of NPIs was estimated to result in recurrent epidemic surges in almost every US state while a delay of even 1 month was estimated to result in marked reductions to the peak of the mortality curve and the burden of severe COVID-19 illness on US hospitals (Linas et al., 2022).

There is robust evidence regarding the effectiveness of NPIs, especially social distancing, in controlling the spread of SARS-CoV-2 (Lai et al., 2020, Ng et al., 2021). Although extreme public health interventions, like stay-at-home mandates, and closure of non-essential activities were initially critical to flattening the curve and limiting dispersion of transmission (Lai et al., 2020, Ng et al., 2021), it was the layered, continuous, and long-term implementation of additional strategies that contributed to mitigate SARS-CoV-2 transmission in Merida. Given the very high transmissibility of the delta (B.1.617.2) variant and the heterogenous efficacy of vaccines, the opportune and continued implementation of NPIs may be necessary to control community transmission and avoid overwhelming health-care systems even with vaccine coverages as high as 80% (Leung et al., 2021). If interventions in China had been implemented one week, two weeks or three weeks earlier than they actually were, the number of cases of COVID-19 could have been reduced by 66%, 86% or 95%, respectively and the geographical range of affected areas would have been reduced from 308 cities to 192, 130 or 61 cities, respectively (Lai et al., 2020). On the other hand, if NPIs had been introduced one, two or three weeks later than they were, the number of cases might have increased by 3-fold, 7-fold or 18-fold, respectively (Lai et al., 2020). Suppression strategies have achieved good results, but the maximum potential impact of the actions may be lost if those measures are lifted before a real control of the epidemic is established. Mobility data can be used to estimate when and where persons are congregating, a precondition for transmission, but do not sufficiently capture behaviors such as mask wearing, physical distancing, or moving activities outside.

We acknowledge several limitations to our study. First, our results were based on parameters that were estimated for symptomatic cases identified and diagnosed during the outbreak, and do not account for asymptomatic and mild infections; we may therefore have underestimated the total number of infections. Assuming that at the city level symptomatic and asymptomatic cases followed a similar temporal trend, our estimates of Rt would not be significantly biased by the use of reported cases only as they represent a relative measure of transmission intensity. Finally, an central to our evaluation of NPIs, the fact that all interventions were layered in combination prevented us to estimate their impact in isolation. Furthermore, and as a result of this being an observational study, the absence of a ‘control’ group of individuals prevented us from making more robust estimates of NPI efficacy and impact.

## 5 Conclusions

Our study shows that population dynamics influenced the transmission of the novel SARS-CoV-2 and the trend of the disease in a large tropical urban center. A controlled and planned ease of restrictions to balance health, social and economic recovery resulted in a single wave of transmission that prolonged at low and stable Rt levels and that prevented overcrowding of intensive-care units and the entire healthcare system. The lockdown of the capital city did not halt the spread of the virus but contributed to the containment of transmission to manageable levels for the public health system. Our results highlight that even in countries like Mexico, plans for rapid pandemic response involving multiple interventions should be implemented (particularly NPIs at the beginning of an outbreak) to more effective and rapidly contain transmission and reduce the size of the outbreak. Reducing contact and increasing social distance between individuals including gathering size restrictions (business and restaurant restrictions and social gatherings), event size restrictions, and overall lockdown/stay at home orders as well as alcohol sales), together with improved personal hygiene (mask mandates), helped protect vulnerable populations and mitigated the spread of COVID-19 in Yucatan, and these interventions should be promoted to contain future waves or COVID-19 or any potential pandemic with similar mode of transmission.

## Funding

GVP received funding from Emory University through the MP3 Initiative (Vazquez-Prokopec and Collins M, co-PI). PMS received funding from IDRC (Project 109071-002).

## Data sharing statement

Data used in this article was derived from administrative health and social data as a secondary use. The original source data is not owned by the researchers and as such cannot be provided to a public repository.

## Evidence before the study

Mexico is the third worst affected country in America by the COVID-19 pandemic. Limited information is available on the implementation of Non-Pharmacological Interventions (NPIs) in Mexico and the effect on the transmission dynamics of the pandemic at national or regional level.

## Added value of this study

The implementation of NPIs has significantly affected the evolution of the pandemic worldwide. However, the socioeconomical context of each affected country was the main driver on duration, timing, periodicity, and adherence to the NPIs. Mexico was not the exception, and each of the 32 states responded locally to the pandemic with a mix of heterogeneous NPIs. Yucatán was the state that implemented an array of NPIs, including alcohol sales ban and one of the longest and sustained stay-at-home mandates in Mexico. We analyzed the effect of fourteen local and federal NPIs implemented by the Yucatan government at four different intervals of time during the first year of the COVID-19 pandemic. We show that in Yucatan, Mexico the implementation of layered NPIs contributed to an economic recovery within a single wave of transmission at low and stable levels. The Rt indicator was reduced through long sustained adherence to NPIs which helped decrease human mobility and contact patterns throughout geographical zones in the metropolitan area.

## Implications of all the available evidence

Understanding the COVID-19 pandemic under the context of country socioeconomic and demographics are crucial to minimize the costs of their interventions.

## CRediT authorship contribution statement

**G. Ayora-Talavera:** Conceptualization, Supervision, Writing – original draft, Writing – review & editing. **P. Granja-Perez:** Resources. **M. Sauri-Vivas:** Resources. **C.I. Hernández-Fuentes:** Resources. **I.P. Hennessee:** Data curation, Formal analysis, Methodology. **I. López-Martínez:** Resources. **G. Barrera-Badillo:** Resources. **A. Che-Mendoza:** Data curation, Formal analysis. **P. Manrique-Saide:** Conceptualization, Funding acquisition, Writing – original draft, Writing – review & editing. **J.A. Clennon:** Data curation, Formal analysis, Methodology. **H. Gómez-Dantés:** Writing – original draft, Writing – review & editing. **G. Vazquez-Prokopec:** Conceptualization, Data curation, Funding acquisition, Methodology, Supervision, Writing – original draft, Writing – review & editing.

## Declaration of Competing Interest

The authors declare that they have no known competing financial interests or personal relationships that could have appeared to influence the work reported in this paper.
